# The influence of city development on urban pedodiversity

**DOI:** 10.1038/s41598-022-09903-5

**Published:** 2022-04-09

**Authors:** Sylwia Pindral, Rafał Kot, Piotr Hulisz

**Affiliations:** 1 Faculty of Earth Sciences and Spatial Management, Department of Soil Science and Landscape Management, Nicolaus Copernicus University in Toruń, Lwowska 1, 87-100 Toruń, Poland; 2 Faculty of Earth Sciences and Spatial Management, Department of Geomorphology and Quaternary Paleogeography, Nicolaus Copernicus University in Toruń, Lwowska 1, 87-100 Toruń, Poland

**Keywords:** Environmental sciences, Environmental impact

## Abstract

The aim of this study was to use a pedodiversity index (PI) to assess changes in the spatial structure of soil cover in Inowrocław, Poland during the twentieth and twenty-first centuries. An original cartographic approach based on landscape metrics was implemented using GIS techniques and statistical calculations. Based on maps of urban soil complexes and pedodiversity, it was revealed that land and soil cover changes in two studied periods (1934–1978 and 1978–2016) significantly affected pedodiversity in the city. In general, the spatio-temporal increase of the pedodiversity index was observed. The percentage of highest values of the PI ranged from 15.9% in 1934, 17.3% in 1978 to 20.9% in 2016. We revealed that pedodiversity index (PI) values are highly spatially and temporally variable and are associated with urban development and changes in the city’s internal structure. The applied approach allowed for the identification of both spatial patterns of changes in soil cover that closely reflect the successive stages of the city's development, and problem areas that require revitalization and the implementation of the principles of sustainable development. Therefore, the proposed method can be recommended for landscape monitoring and in determining ecosystem services in urban and landscape planning, and environmental management.

## Introduction

The dynamic population growth of recent years^1^ has had numerous environmental consequences that can negatively affect both the natural environment and the quality of human life^2^. Many changes resulting from the increase in global population are caused by the need to acquire new areas for housing and associated commercial areas, industry and transport networks. Considering the spatial approach to this problem, the phenomenon of urban sprawl is defined as the expansion of built-up areas onto land adjacent to the city^3^. This is accompanied by the development of related infrastructure. This results in undeveloped or slightly built-up areas being transformed into high-density residential areas. Environmentally, this has several unfavourable consequences, such as increasing the degradation of semi-natural areas or reducing biodiversity^3–5^. For this reason, monitoring temporal and spatial changes within semi-natural, anthropogenic areas and changes in landscape diversity plays an important role in environmentally sustainable management and development^6,7^.

Urban soils are created by human activity in the process of city development^8–10^. Hence, their properties and functions can differ from those of semi-natural soils. The pedogenesis of urban soils depends on the deposition of anthropogenic and technogenic materials and the intensity of their transformation^11–13^. Since the acreage of anthropogenic and technogenic soils in the city has increased significantly, the importance of the function of urban soil in the environment is being emphasised. As reported by Liang et al.^14^, Morel et al.^15^, and Sallustio et al.^16^, urban soils can provide a wide range of ecosystem services such as food and biomass production, dilution, filtration, sequestration of pollutants and nutrient resources, storage of genetic materials, and use for recreation, information and knowledge.

One of the essential tools for sustainable management and development of urban soils is soil mapping. Because the genesis of urban soils is connected with human activity, soil maps within cities are based mostly on land-cover maps^17–19^. Considering the high heterogeneity of urban soils and the strong fragmentation of the soil cover^20^, there is a need to apply an approach that groups urban soil types by their function, genesis or properties. For this purpose, urban soil complexes—or urban pedotopes—may be applied in urban soil cartography^21,22^ as well as other spatial units having specific abiotic, biotic and socio-economic characteristics specifying the soils' properties^23^.

Pedodiversity analysis is a method for assessing urban soil cover both in space and time. It is described as the variation of soil properties, units or types within an area^24^. Research on soil diversity is quite wide-ranging, and uses various methods^25–27^. Landscape metrics are the most common indices used in pedodiversity evaluation^14,22,28^. They allow a quantitative analysis of the spatial structure of the landscape. Soil diversity research has been undertaken repeatedly in recent years^25,27,29,30^. However, comprehensive studies on urban soil diversity^22,31^ are still very rare, especially those focused on temporal changes in the structure of the soil cover.

In this study, we used a pedodiversity index (PI)^22^ to assess changes in the spatial structure of soil cover in the city of Inowrocław, Poland during the 20th and 21st centuries. An original cartographic approach based on landscape metrics was implemented using GIS techniques and statistical calculations. This city was selected for the study due to the potentially high soil heterogeneity within a relatively small area^22^. The objective was accomplished by several research tasks, namely: (i) application of the qualitative-quantitative method developed by Pindral et al.^22^ to visualise changes related to the city’s development that have occurred within two time intervals: 1934–1978 and 1978–2016, (ii) identification of spatial patterns in the urban pedodiversity and (iii) explanation of the factors influencing the direction of changes in the spatial structure of the city and its impact on the soil cover. It was hypothesised that the pedodiversity index (PI) can be a useful indicator of anthropisation, i.e. city development. Diversity analyses help to understand the spatial structure of the city by officials, landscape architects, spatial planners, and thus can complement the designation of areas requiring rehabilitation and improving the city's aesthetic and utility values.

## Materials and methods

### Study area

**Figure 1:**
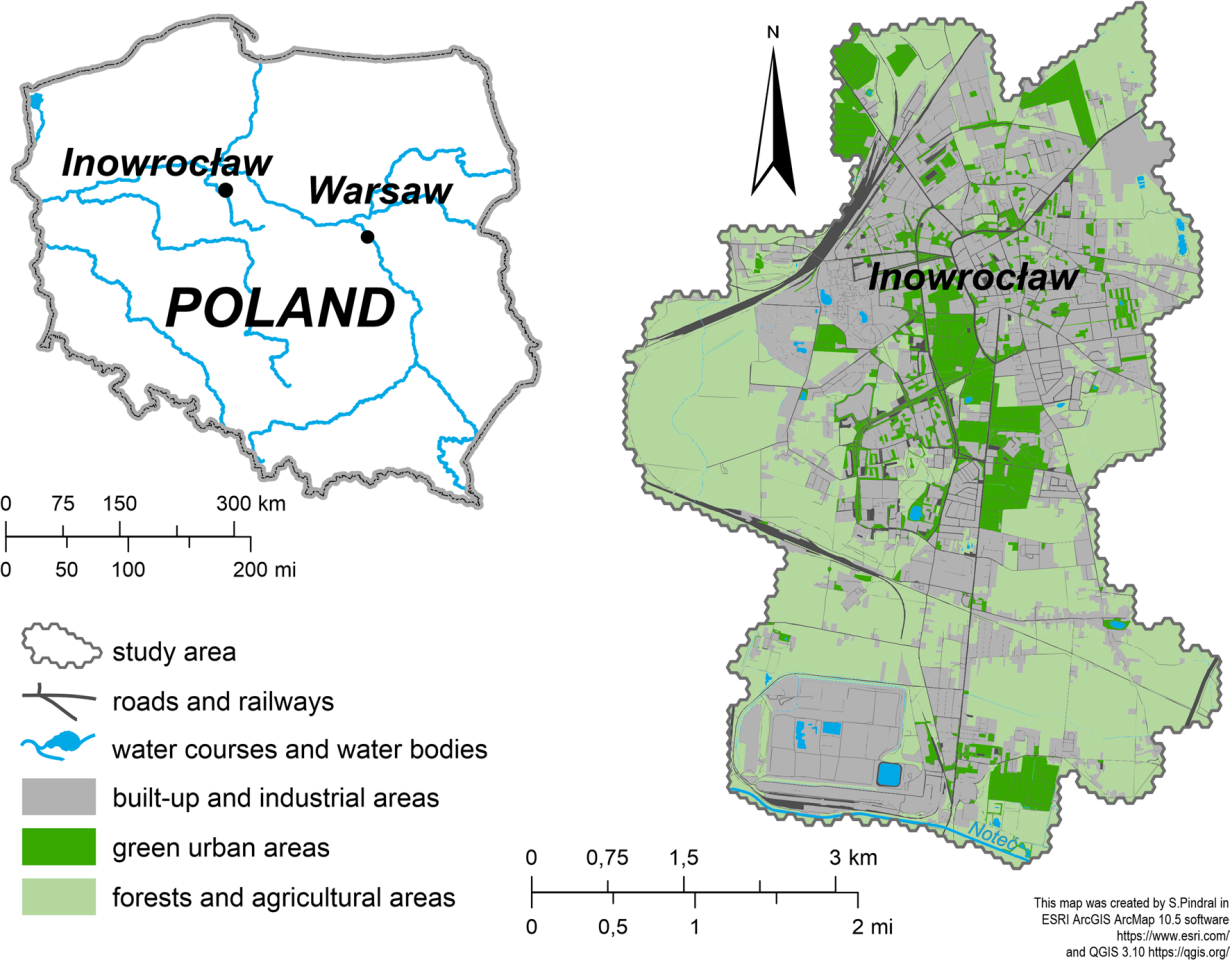
Location of study area.

Inowrocław, founded in 1238 is one of the oldest cities in Poland. This medium-sized city (30.42 km^2^) of 71,674 people is located on the Inowrocław Plain^32^ in the Kuyavian-Pomeranian voivodeship of north-central Poland (52°47′45″N, 18°15′40″E**)** (Fig. 1). It is a territorial unit with one of the highest population densities in Poland, at 2356 people per km^2^, compared to a national average of 122 (status for 2020, bdl.stat.gov.pl).

The relief within the city area was ultimately formed during the Weichselian glaciation and Holocene and is mostly situated within a glacial till plain covered by Mollic Gleysols and Gleyic Phaeozems^33^. Natural factors led to the delimitation of four variations of natural landscapes within the study area: flat morainic plains with agricultural land, undulating morainic plains with agricultural land, a flat floodplain with agricultural land, and overflood erosive-accumulative terraces, forestless with wasteland^34^. The city centre is located on the culmination of the Permian salt dome which has had an important influence on the environment conditions. Mineral waters associated with shallow salt deposits may contribute to the salinity of soils and thus change their nutritional properties^35^.

For many centuries, Inowrocław was an important administrative city and trade centre^36^. The economic development of the city began under the Prussian partition, mainly in the 19th century. At that time, the chemical industry, railway transport and a health resort began to develop. After restoring independence, the city’s economic development continued, with such activities as glass industry, rock salt mining and a spa. In the 20th century, the city’s growth resulted in the incorporation of neighbouring villages. After World War II, further economic development of the chemical and machine industries, mining and metallurgy took place in Inowrocław^36,37^. This caused the rapid environmental changes resulting in agricultural land losses and degradation of natural habitats, especially meadows and pastures. At the end of the 20th century, many industrial plants collapsed, and the industry was restructured. Nowadays, Inowrocław is a multifunctional city (industry, agriculture, transport, spa).

### Data sources

**Figure 2:**
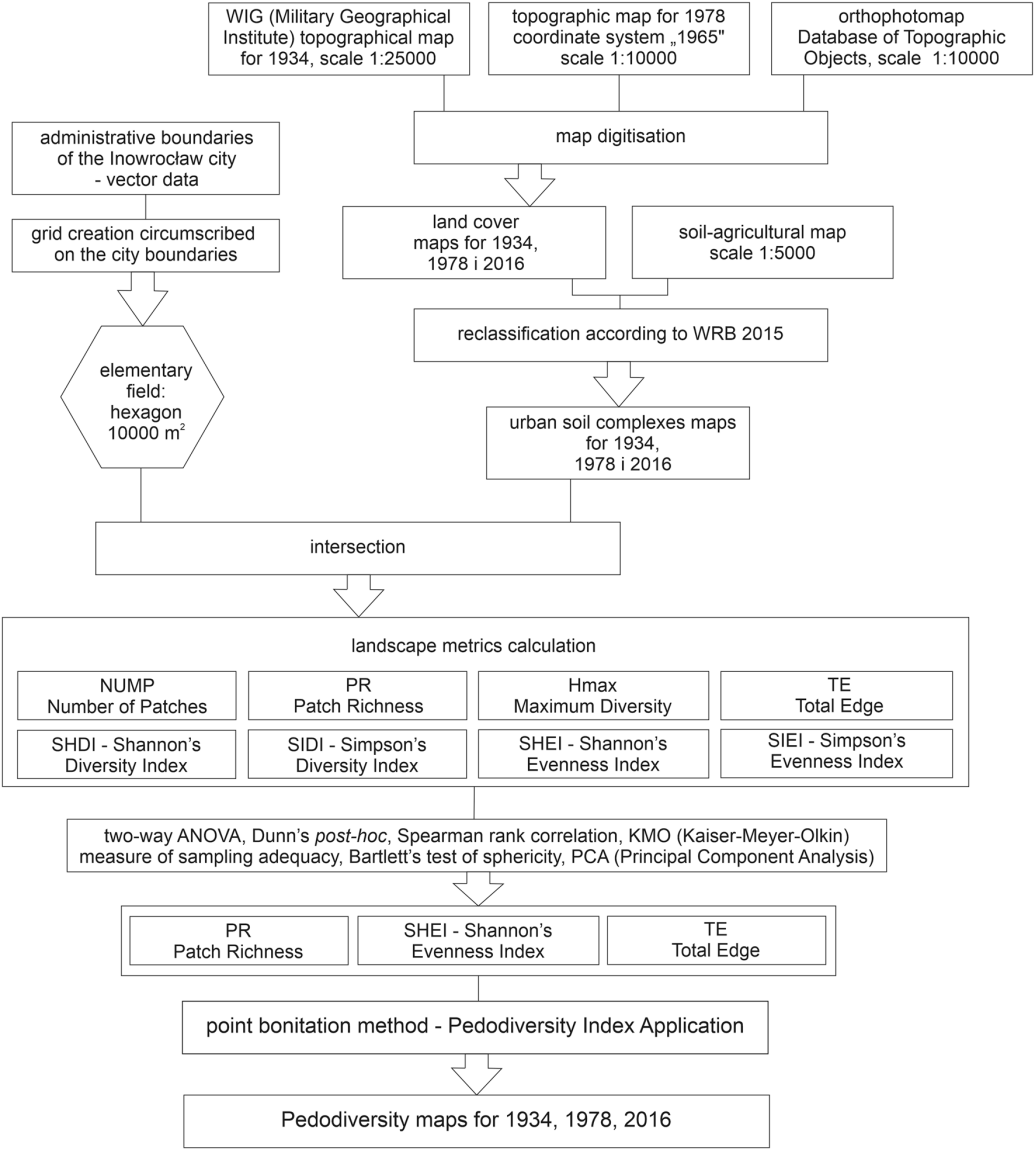
A flow chart of research methodology.

Taking into account the availability and thematic consistency of basic cartographic materials, we decided to use three maps from 1934, 1978, and 2016 (Fig. 2). The oldest available detailed map of Inowrocław was the topographic map of the Military Geographical Institute updated in 1934 at a scale of 1:25,000 (http://igrek.amzp.pl/). Another source material was a topographic map developed in the "1965" coordinate system at a scale of 1:10,000. The map was updated for 1978 (http://mapy.geoportal.gov.pl/imap/). The maps for 2016 were developed using orthophoto maps and a detailed Database of Topographic Objects. In addition, a soil-agricultural map at the scale of 1:5000 was used for all analysed periods to identify and classify undisturbed or weakly transformed soils (http://geoportal.infoteren.pl/kompozycje.html; Fig. 2)^22^. The soil-agricultural maps present the historical record of the state of the soil cover in Poland, i.e. from the mid-20th century, especially in the context of soils of organic origin and agricultural soils in areas exposed to water and aeolian erosion. That is why the soil map units have been reinterpreted and reclassified in accordance with the criteria proposed by Świtoniak et al.^38^.

### Grid creation and map digitisation

The pedodiversity analysis was done within a grid of artificial units—elementary fields. For this purpose, a grid of 3205 interlocking equal hexagons was created using MMQIS plugin (https://plugins.qgis.org/plugins/mmqgis/). The size of each elementary field was chosen based on similar research^22,39^ and calculations according to the map's scale following the Hengl^40^. The most suitable surface area of the elementary field is 10,000 m^2^^40^ and the most convenient shape for grid creation was a hexagon. One of the biggest advantages of the hexagonal grid is that each element is closer to a circle than the square (considering shape), thus there is lower anisotropy with hexagons. Another is that the distance between centroids is the same in all six directions. In contrast, distance to neighbours at the corner and at the side of square cells varies. These properties provide more accurate sampling using hexagonal grid. Considering this, the final grid was comprised of equilateral hexagons^41^. The map grid was delimited by the administrative boundaries of the city. Within the boundaries of the created grid, two archival topographic maps (for 1934 and 1978) were digitized. Land cover categories were created using these maps following the Corine Land Cover 2018^42^ classification (Table 1).

**Table 1:** Relationships between land cover classes, WRB soil units and urban soil complexes (USCs)^22^.

Land cover classes	Urban soil complexes (according to IUSS Working Group WRB, 2015)	
	Code	Soil map units
Forests, pastures, meadows, arable lands	USC 1	Mollic Gleysols, Gleyic Phaeozems
Forests, pastures, meadows, arable lands	USC 2	Gleyic Luvisols (Aric, Colluvic), Stagnic Luvisols (Aric, Colluvic), Brunic Arenosols, Murshic Histosols, Hemic/Sapric Histosols
Parks, gardens, lawns, allotments	USC 3	Hortic Anthrosols, Mollic Gleysols (Technic), Gleyic Phaeozems (Technic), Hortic Phaeozems
Cemeteries and graveyards	USC 4	Gleyic Phaeozems (Relocatic), Ekranic Technosols
Dirt roads	USC 5	Technosols (Densic, Transportic)
Pavements, main roads, squares, car parks	USC 6	Ekranic Technosols
Industrial or commercial area, waste ponds, railway	USC 7	Spolic Technosols (Salic, Sodic), Mollic Gleysols (Technic), Mollic Gleysols (Salic, Sodic), Garbic Spolic Technosols (Humic)
Continuous urban fabric, discontinuous urban fabric	USC 8	Urbic Technosols, Mollic Gleysols (Technic)
Rivers, ponds, lakes	Water bodies	

### Urban soil classification

The new urban soils maps were made based on data obtained from land cover and soil-agricultural maps. These two maps were intersected with each other. The resulting shapefile was reclassified according to the units from both maps (land cover classes and soil units). Considering the high heterogeneity and fragmentation of the urban soil units, urban soil complexes (USCs) were distinguished as soil units with similar use, degrees of human transformation and ecosystem services^22^. They were correlated with WRB soil classification system^33^ (Table 1).

### Landscape metrics calculation

The chosen metrics have been applied most frequently for quantitative diversity analysis^14,22,25,39^. Based on the urban soil complexes map, for each field of the hexagonal grid we calculated selected landscape metrics. The calculations were based on the FRAGSTAT^43^ formulas and were applied for each of the 3205 elementary fields. Pedodiversity analysis can encompass many metrics, but in our research we used classic metrics based on the area, number and shape of patches observed in the spatial structure of urban soil complexes. Thus, eight landscape metrics were calculated according to McGarigal and Marks^43^, Shannon and Weaver^44^, and Simpson^45^

#### Number of patches (NUMP)^43^

$$NUMP=n$$

where *n* is the number of patches occurring in the landscape.

#### Patch richness (PR)^43^

$$PR=m$$

where *m* is the number of classes (USCs) occurring in the landscape.

#### Total edge (TE): the length of the boundaries within the hexagon^43^

$${\text{TE}} \ge 0$$

TE = 0, for the hexagon includes only one polygon (PR = 1).

#### Maximum diversity (Hmax)^43^

The measure calculates maximum possible landscape diversity, the state when all classes (m) occur in the landscape in equal proportion.

$$Hmax=ln(m)$$

$${\text{Hmax}} \ge 0$$

#### Shannon’s diversity index (SHDI)^43,44^

$$SHDI=-{\sum }_{i=1}^{m}({P}_{i}\times ln{P}_{i})$$

where Pi is the proportion of the landscape occupied by the type of class.

#### Shannon’s Evenness Index (SHEI)^43,44^

$$SHEI=\frac{-{\sum }_{i=1}^{m}({P}_{i}\times ln{P}_{i})}{lnm}$$

$$0 \le {\text{SHEI}} \le {1}$$

#### Simpson's Diversity Index (SIDI)^43,45^

$$SIDI=1-{\sum }_{i=1}^{m}{P}_{i}^{ 2}$$

$$0 \le {\text{SIDI}} \le {1}$$

#### Simpson's Evenness Index (SIEI)^43,45^

$$SIEI=\frac{1-{\sum }_{i=1}^{m}{Pi}^{2}}{1-\frac{1}{m}}$$

$$0 \le {\text{SIEI}} \le {1}$$

SHDI, SHEI, SIDI, SIEI = 0 when the landscape contains only one class (PR = 1). When these values approach 0, the distribution of the area between the different soil types within the hexagon becomes increasingly irregular (i.e., dominated by one type)^43^.

### Statistical validation and pedodiversity index calculation

Before the landscape metrics calculation, to assess whether the results of the calculated indexes are appropriate for the application of Principal Components Analysis (PCA), a few steps were taken on the procedure presented in Fig. 2. To assess whether the data are statistically significant and to measure the variability of calculated metrics, two-way ANOVA was applied with the Kruskal–Wallis test for equal medians and Dunn's post-hoc analysis^30,46,47^ for the three analysed periods. The calculations clearly showed a significant difference among sample medians; hence, we can include all metrics in the PCA (Supplement 1). The obtained results are the same for each analysed year. The results of the ANOVA were confirmed by Bartlett’s test and Kaiser–Meyer–Olkin (KMO) measure of sampling adequacy. The value of KMO is higher than 0.5 and the significance level in Bartlett's test is less than 0.05, so a factor analysis is useful with the input data^22,48^ (Supplements 1 and 2). Both measures indicate that all landscape metrics are related.

The last step before the calculation of the PI equation is to simultaneously analyse the results of Spearman's rank correlation and PCA. The purpose of these calculations is to exclude from the final pedodiversity index all metrics that are highly correlated with each other and thus do not explain the diversity phenomena as well as less correlated ones. In this method the inclusion of other metrics and variables potentially linked to the pedodiversity is possible. This step of statistical validation allows us to reduce the calculated metrics and to select the most relevant. The highest loading on the first axis was assigned to PR for all periods. The least correlated with PR are TE (Total Edge), SIEI (Simpson’s Evenness Index), and SHEI (Shannon’s Evenness Index) (<0.8) for 2016 and 1978. For 1934, the following metrics are more correlated (>0.8), yet SHEI, TE, SIEI and SIDI were the least correlated with PR (Supplement 1). After Olivieri^49^ we can take into account loadings from three axes calculated in PCA. On the second axis, the highest value that was assigned was SIEI; on the third axis, the largest loading was for TE. To sum up, after considering Spearman's rank correlation and PCA, three landscape metrics were included in the PI calculation: PR, SIEI and TE. Their dissimilarity was validated by a classification tree. PR, SIEI and TE have relatively long distances, thus they are the least related to each other according to their values distribution (Supplement 2). The application of the bonitation method involved dividing each landscape metric values into four class intervals using Jenks natural breaks method^39^. The number of intervals was taken from previous work^22^ to provide fully comparable data of changes in soil diversity for the analysed periods. For each class, points (ranks) were assigned – from 1 (the lowest values) to 4 (the highest values). The last part of the pedodiversity analysis involved calculating the modified pedodiversity index (PI), which was proposed by Pindral et al.^22^ using the point bonitation method^50^ according to the following formula:

$$PI=PRr+TEr+SHEIr$$

where: PRr—rank of Patch Richness ranges, TEr—rank of Total Edge ranges, SHEIr—rank of Shannon’s Evenness Index ranges.

The pedodiversity index (PI) ranges from 3 to 12, where 3 is the value occurring within the least diverse hexagons (with only one patch) and 12 characterised the places with multiple USCs and thus with the biggest fragmentation of the soil cover.

## Results

### Soil cover changes

**Figure 3:**
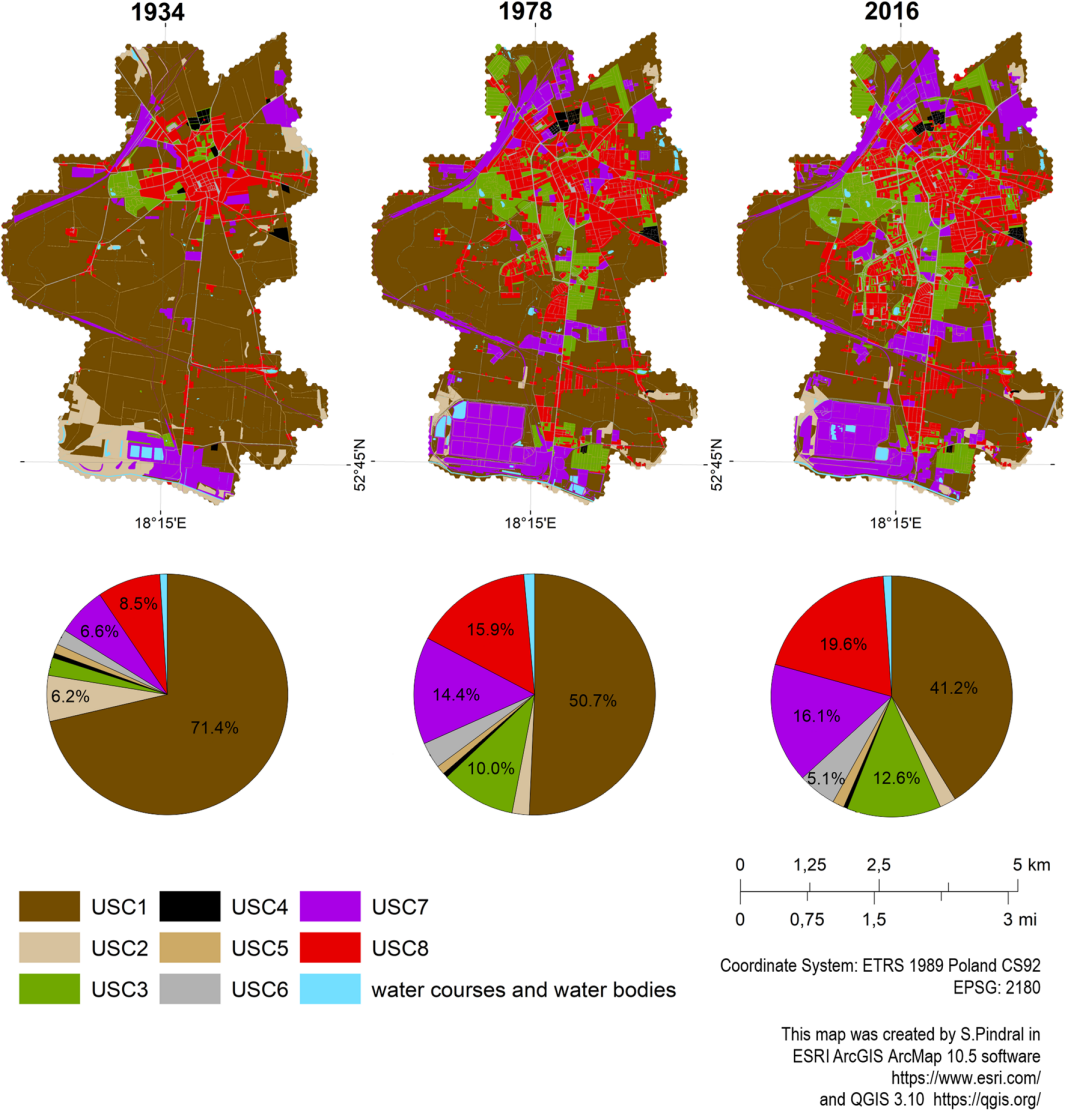
Maps of urban soil complexes (USCs) for selected years.

In the analysed period 1934–2016, the spatial structure of the city changed significantly (Figs. 3, 1). Most of the calculated descriptive statistics for both land cover and urban soil complexes patches decreased period by period (except the maximum value and range) (Supplement 3: Table I). The area of the most transformed soils increased, i.e. Urbic Technosols, Mollic Gleysols (Technic) (USC8: 1934—8.5%, 1978—15.9%, 2016—19.6%), Spolic Technosols (Salic, Sodic), Mollic Gleysols (Technic), Mollic Gleysols (Salic, Sodic), Garbic Spolic Technosols (Humic) (USC7: 1934—6.6%, 1978—14.4%, 2016—16.1%); and Ekranic Technosols (USC6: 1934—2.1%, 1978—3.5%, 2016—5.1%). As a result, weakly transformed soils, mainly Mollic Gleysols and Gleyic Phaeozems (USC1 and USC2) decreased significantly (1934—77.6%, 1978—53.0%, 2016—43.4%). For the other USCs, no significant changes were observed. The spatial structure of the soil cover within Inowrocław changed according to land cover changes radially from the city centre to the administrative boundaries (Fig. 3). According to the built-up area expansion, new patches of transformed soils appeared. Most USC4 was transformed into USC1 in the period 1934–1978, while in the period 1978–2016 it was transformed into USC5 and USC6. In turn, USC5 underwent some major changes in 1934–1978 to USC1 and USC2, while in the period 1978–2016 the area was transformed into USC1, USC3 and slightly into USC6, USC7 and USC8. The spread of USC8 occurred from the city centre radially outwards. Formerly discontinuous USC8 patches were transformed into more densely clustered patches. Subsequently, USC7 expanded between 1934 and 1978, but for 2016 the area of USC7 was fragmented and some of these urban soil complexes changed into USC1 and 2, especially in the southern part of the city (Fig. 3; Table 2). In parallel to the increase in USC7 and 8, the area of USC4 and USC6 was also grown (Fig. 3). Another spatial aspect was a large increase in urban green areas—USC3 (1934—0.6%, 1978—10.0%, 2016—12.6%). In 1978, this area grew significantly adjacent to USC8. In 2016, relatively small patches of USC3 appeared within the continuous USC8 area (Fig. 3; Table 2). The last unit—water bodies—is associated with an area increase in the period 1934–78, but with a decline in the period 1978–2016. Water bodies were transformed mainly into agricultural areas (part of the USC1), and in the period 1978–2016 also into USC7 (Table 2).

**Table 2:** USCs area changes between selected years [km^2^].

1934	1978								
	USC 1	USC 2	USC 3	USC 4	USC 5	USC 6	USC 7	USC 8	Water bodies
USC 1	+ 1.7219	0.3738	2.2286	0.0553	0.2402	0.6177	2.1079	2.6061	0.1188
	− 8.3484								
USC 2	0.5836	+ 0.4430	0.0593	0.0008	0.0510	0.0392	0.7014	0.0381	0.2072
		− 1.6817							
USC 3	0.0832	0	+ 2.7794	0.0111	0.0210	0.0377	0.0538	0.1383	0.0136
			− 0.3587						
USC 4	0.0233	0	0.0203	+ 0.0800	0.0070	0.0119	0.0022	0.0087	0.0004
				− 0.0738					
USC 5	0.1787	0.0138	0.0478	0.0024	+ 0.3673	0.0288	0.0528	0.0529	0.0034
					− 0.3806				
USC 6	0.1130	0.0036	0.1006	0.0062	0.0052	+ 1.0101	0.0646	0.2480	0.0016
						− 0.5467			
USC 7	0.4275	0.0424	0.0551	0.0004	0.0247	0.0639	+ 3.3117	0.1704	0.0470
							− 0.8314		
USC 8	0.2215	0.0013	0.2574	0.0039	0.0080	0.2091	0.1942	+ 3.2658	0.0038
								− 0.8992	
Water bodies	0.0862	0.0081	0.0104	0	0.0091	0.0018	0.1347	0.0034	+ 0.3959
									− 0.2547

1978	2016								
	USC 1	USC 2	USC 3	USC 4	USC 5	USC 6	USC 7	USC 8	Water bodies
USC 1	+ 0.9195	0	0.8329	0.0005	0.1802	0.4061	0.8774	1.5834	0.0862
	− 3.9667								
USC 2	0	+ 0.0883	0.0011	0.0002	0.0111	0.0151	0.0702	0.0122	0.0184
		− 0.1283							
USC 3	0.0884	0	+ 1.5755	0.0005	0.0949	0.1708	0.0758	0.2844	0.0063
			− 0.7211						
USC 4	0.0256	0.0002	0.0197	+ 0.0173	0.0023	0.0108	0.0001	0.0029	0
				− 0.0616					
USC 5	0.0655	0.0084	0.1197	0.0031	+ 0.4431	0.0184	0.0819	0.0214	0.0047
					− 0.3231				
USC 6	0.0723	0.0086	0.0989	0.0088	0.0482	+ 1.0394	0.0859	0.1956	0.0002
						− 0.5185			
USC 7	0.3424	0.0217	0.0948	0.0023	0.0693	0.1006	+ 1.5123	0.2479	0.0927
							− 0.9717		
USC 8	0.2321	0.0057	0.3976	0.0019	0.0338	0.3109	0.1731	+ 2.3517	0.0013
								− 1.1565	
Water bodies	0.0932	0.0436	0.0108	0	0.0032	0.0067	0.1480	0.0039	+ 0.2097
									− 0,3094

### Pedodiversity changes

**Figure 4:**
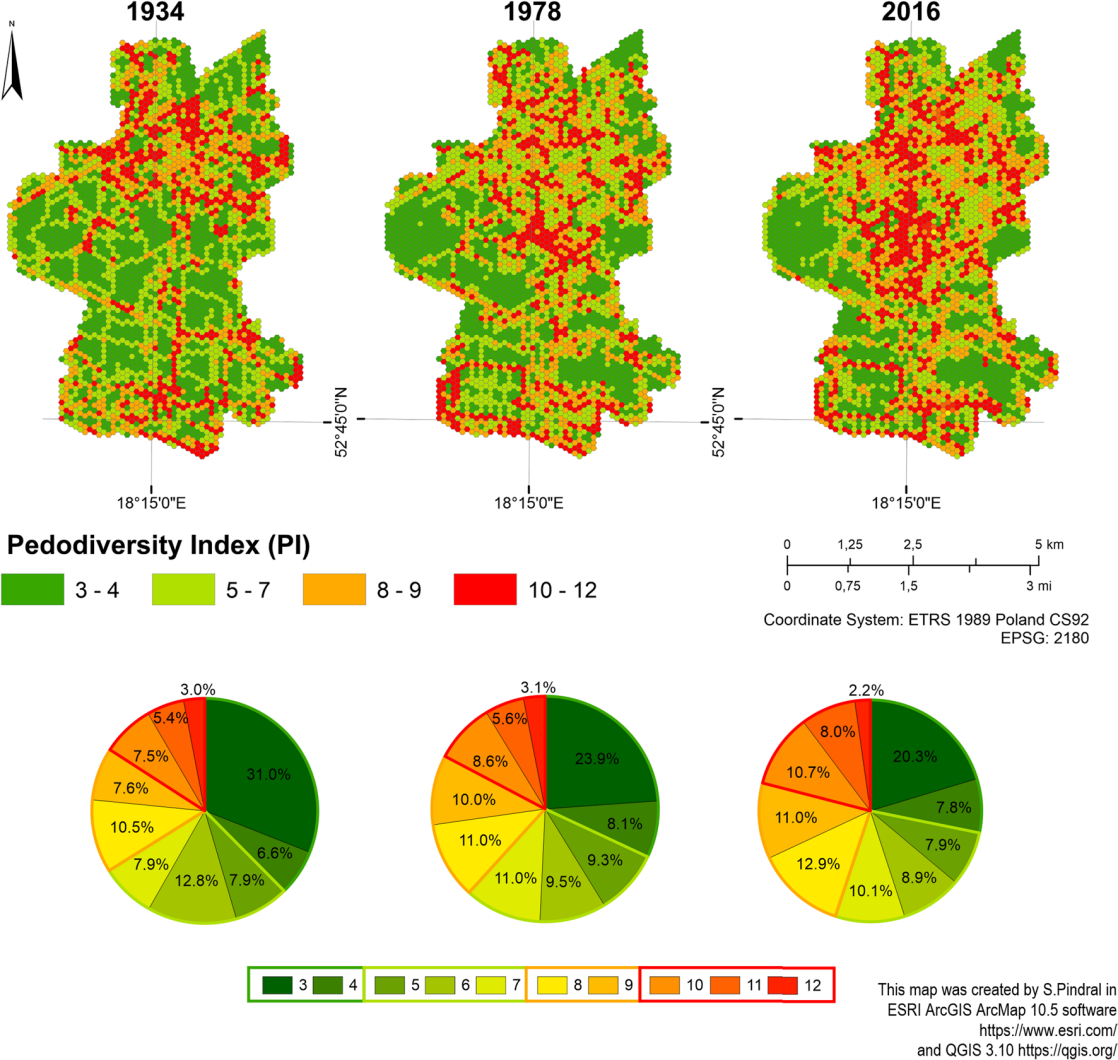
Pedodiversity index maps for selected years.

In the analysed periods, there were clear trends in quantitative and spatial changes in soil diversity. All the calculated landscape metrics median, mean, standard deviation, the first and the third quartile increased period by period. The only exception was PR and Hmax values which had the same range in 1978 and 2016 (see Supplement 3, Table II). In the range of PI values from 3 to 4, as the city grew, the number of hexagons with the lowest variety decreased. The next range (PI 5–6) showed no significant trends, but the extent of the high (8–9) and the very high (10-12) PI values was remarkably enlarged as the city developed. For the range of PI values from 8 to 9, the share of hexagons increased from 18.1% in 1934, through 21% in 1978, to 23.9% in 2016. In the case of the highest values (PI 10-12), their share increased from 15.9% in 1934 to 17.3% in 1978, to 20.9% in 2016 (Fig. 4; Table 3). Similar relations were visible for the share of individual PI values across the entire city, with the exception of the values of 4, 5, 7 and 12, which occurred in greatest numbers in 1978. Considering spatial changes, the greatest changes in soil diversity occurred between 1934 and 1978. For 1934–1978 and 1978–2016 alike, the biggest differences in PI values were observed in the city centre and areas of intensive industrial development in the south of Inowrocław (Fig. 4; Table 3; Supplement 3: III).

**Table 3:** Number and share of hexagons within study area.

PI value	2016		1978		1934	
	Number of hexagons	Share [%]	Number of hexagons	Share [%]	Number of hexagons	Share [%]
3	652	20.34	765	23.87	993	30.98
4	251	7.83	259	8.08	211	6.58
5	254	7.93	297	9.27	252	7.86
6	285	8.89	305	9.52	411	12.82
7	324	10.11	353	11.01	250	7.80
8	415	12.95	355	11.08	337	10.52
9	351	10.95	320	9.98	242	7.55
10	344	10.73	275	8.58	240	7.49
11	257	8.02	180	5.62	172	5.37
12	72	2.25	96	2.99	97	3.03
Total	3205	100	3205	100	3205	100

**Figure 5:**
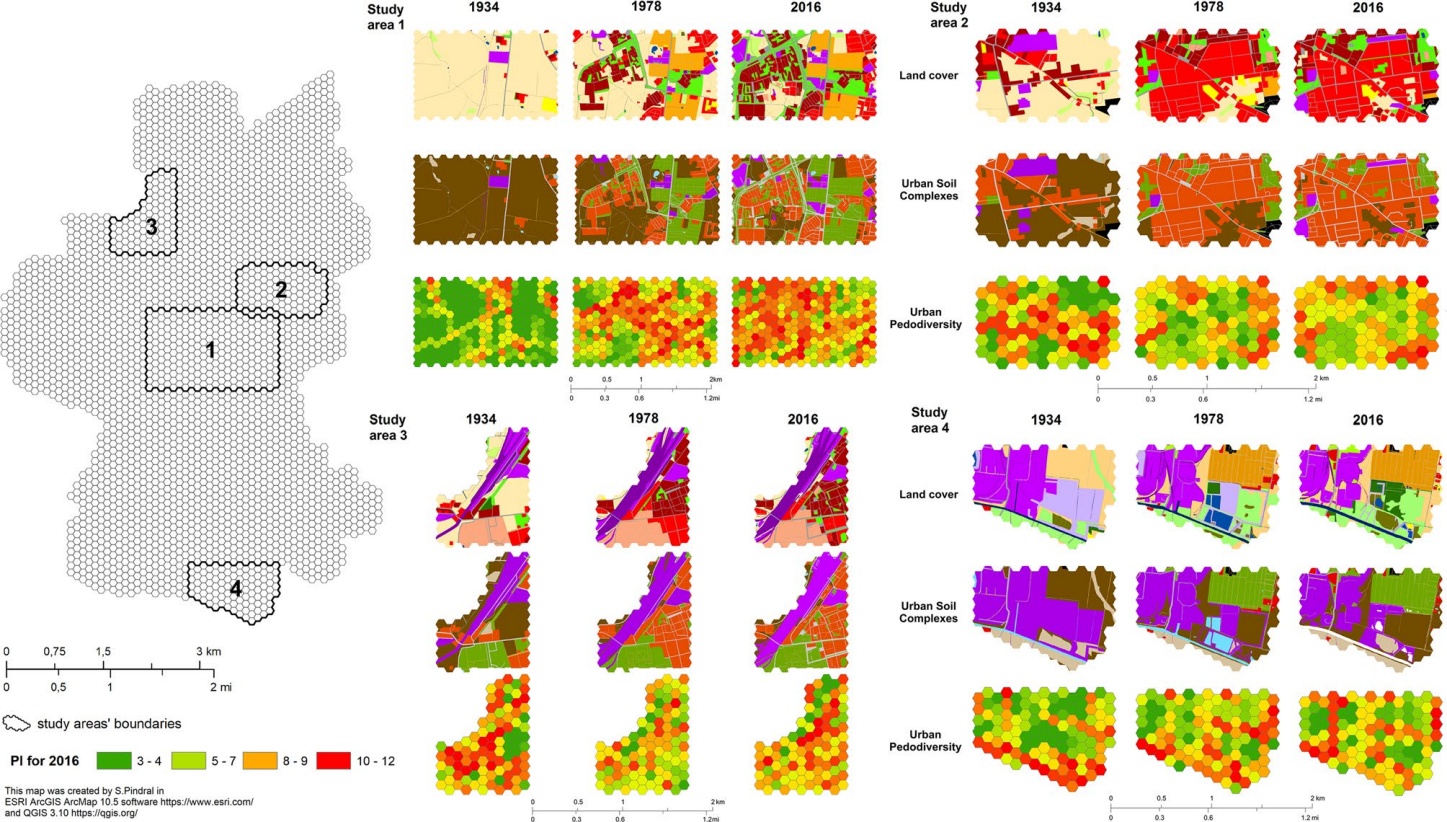
Patterns of urban pedodiversity changes according to land cover and urban soil complexes’ development.

Based on the detailed city map analysis, four general patterns of urban pedodiversity changes were identified. They were characterised on the basis of selected examples (Fig. 5).

*Pattern 1* The increase of PI values along with the increase of acreage of strongly transformed soils and loss of arable land (development of discontinuous urban fabric). It was the most common pattern found for all two studied periods (Fig. 5, Study area 1).

*Pattern 2* The decrease of PI values in small areas along with the increase of building density (clusterization of USC8). This pattern was also observed in the city centre (Figure 5, Study area 2).

*Pattern 3* The decrease in PI values in industrial and residential areas due to the clustering of patches between 1934 and 1978, followed by an increase in PI values due to development of the road network (USC6) and green urban areas (USC3) between 1978 and 2016 (Fig. 5, Study area 3).

*Pattern 4* A general decline in the value of PI with the development of industry. However, changes within industrial areas may also result in an increase in fragmentation due to the greater share of green urban areas (USC3) and the development of road infrastructure (USC 6) and single-family housing (USC 8) (Fig. 5, Study area 4).

## Discussion

Recent studies showed that there are different approaches for comprehensive urban soils mapping. However, the combination of the land cover and basic information about natural soils are still the most useful for this purpose^19,22,51,52^. The statistical results for both studied periods are similar and comparable to the studies by Pindral et al.^22^. Additional methods—ANOVA and Ward's classification tree—support selection of least correlated metrics and thus they are the most useful for the pedodiversity index (Supplement 1). Metrics highly correlated with each other represent redundant information and can influence the final result of the pedodiversity index (PI). Thus, the reduction of variables and data structure validation is statistically significant in spatial analysis. Methods using several landscape metrics simultaneously are rarely used^14^, but the data reduction methods are similar^28,53^. So far, to calculate pedodiversity, of the numerous landscape metrics, those most frequently used have been Hmax, SHDI and PR^25,28,54^. According to Pindral et al.^22^, the method presented in this research fully allows for an objective, qualitative-quantitative assessment of pedodiversity, simultaneously including three different landscape metrics. However, when using land cover maps, particular attention should be paid to the scale-related and source selection-related problems resulting from the heterogeneity of urban areas, as indicated by^19,55,56^.

A characteristic feature of urbanised areas is large, multi-layered and dynamic heterogeneity^57^. Such components of urban ecosystems as biotic, physical, social and built complexes can be heterogeneous and significantly affect soil cover^58^. In cities, anthropogenic and technogenic soils usually form a specific mosaic with other less transformed soils^22,51,52^. A high spatial soil diversity is the result of area, linear and point human interference in the natural environment, closely related to the historical and current use of the land^8,20,59^. As shown in our analysis done for 1934-2016 period, the greatest changes in soil cover of the Inowrocław city were mainly related to the transformation of areas with most fertile soils (Mollic Gleysols and Mollic Phaeozems, USC1-2) into land intended for purposes other than agriculture, i.e. buildings, industry and urban greenery (USC3-8). As a result, the total loss of the agricultural land exceeded 14 km^2^, which is almost 1/3 of the present city area (Fig. 3). In general, our results showed that soil diversity expressed as pedodiversity index (PI) increased from 1934 to 2016. However, we identified several relatively small areas where the trend was opposite (Fig. 4; Tables 2 and 3).

The first study period (1934–1978) covered the intensive economic and spatial development of Inowrocław, which took place both after the First World War and the Second World War. It should be emphasised that the city did not suffer any serious damage as a result of both wars^36,37^. The main factor controlling pedodiversity was building of new housing estates for employees and new city residents. This led to a high increase in the share of Ekranic and Urbic Technosols (USC6 and 8), mostly constructed on agricultural sites, and thus also to the significant increase in pedodiversity (Figs. 4 and 5; Tables 2 and 3). However, the reduction in PI values between 1934 and 1978 was observed in some agricultural, residential and industrial areas. Changes of spatial configuration of agricultural land patches within USC1 and USC2 were caused by the growing acreage of large-scale farms (land consolidation and nationalisation after the Second World War), draining of wetlands, and the removal of roads and irrigation channels between fields (Fig. 3). As a result of increasing density of buildings in the city centre, continuous urban fabric patches were formed (USC8 clustering) (Fig. 5; Study area 1). During the first period, the soda industry developed in the southern part of the city. As shown on the map of urban soil complexes (Fig. 3), the industrial land use changes also contributed to shrinking and simplifying of USCs patches, which resulted in drops in PI values.

Between 1978 and 2016, the pedodiversity index (PI) continued increase, but its dynamics was much slower (Figs. 4 and 5; Tables 2 and 3). Most likely, the main limiting factor was the economic crisis in the 1980s and transformational recession including the decline in industrial output and high unemployment after the collapse of Poland's communist regime^60^. In general, the increase in PI values can be explained by the development of housing in the city outskirts and the creation of recreational areas, including allotment gardens. Regardless, the specific development of old residential areas took place. Discontinuous urban fabric was transforming into denser, more continuous fabric, resulting in decreasing fragmentation. This decreased pedodiversity in several zones by combining relatively small patches into homogeneous large-area units (Fig. 5). This was related to the clustering of USC7 and USC8 which group strongly transformed soils like Spolic and Urbic Technosols.

We believe that the presented approach can be useful in urban planning, especially by providing support in identifying problem areas. The qualitative-quantitative method is useful for monitoring soil-cover changes and presents how urban sprawl and the process of urbanisation can influence the soils within a city. The above results revealed that the soil cover in Inowrocław city is highly diverse, and it underwent significant transformations between 1934 and 2016. The pedodiversity analysis using our original pedodiversity index approach undoubtedly allowed the identification of spatial patterns of changes that closely reflect the successive stages of the city's development. Concerning the sustainable development of urban soils, the pedodiversity index and spatial analysis of soil structure within the city may also provide valuable information about the soil transformation and identification of areas where ecologically valuable soils may be endangered. As a result of irrational spatial management and poor allocation of investments (development of commercial, industrial or residential buildings), these decisions may cause soil spatial fragmentation, physicochemical properties changes, and in extreme cases, soil degradation^18,58,59,61^. All those actions can significantly affect the functioning of soils in the ecosystem. Therefore, an appropriate description and quantification of ecosystem services provided by urban soils seems to be very important for human well-being and effective protection of soil resources^62^. It should also be noted that all urban soils, irrespective of the degree of transformation, can provide ecosystem services of high value. According to classification based on the potential of soils of urban, industrial, traffic, mining and military areas (SUITMAs) as vegetation support systems by Morel et al.^15^, it can be stated that currently the largest area of Inowrocław city is occupied by vegetated pseudo-natural (USC1, USC2) and vegetated engineered SUITMAs (USC3, USC 4), and then by bare (USC6, USC8) and dumping SUITMAs (USC7). The first two soil groups have the highest potential for food production and non-food biomass, regulating services (water storage, runoff and flood control, air purification, local climate, biodiversity) and are important for education and recreation. On the other hand, strongly transformed soils with low potential (dumping SUITMAs) may be reservoirs of minerals and archives of human history, and bare SUITMAs may provide some provisioning (fresh water supply) and regulating (pollution attenuation) services. As shown in our study, greater density of buildings and other constructions simplified the spatial distribution of the urban soil complexes, increasing soil homogeneity significantly. It reduced the share of the green spaces that perform such important ecosystem functions in towns and cities^15,62^. However, the phenomenon of urban soil diversity is very complex and sometimes difficult to be unambiguously interpreted^51,63^. For example, if increased diversity is due to the increase of area of the soils affected by sealing (Ekranic Technosols), this could result in significant reductions of soil services^15,18,64^. More diversity is not always good in this context. Low diversity, as if all the soils are in urban greenspace (e.g. forests and agricultural land), is likely to be much better for environmental quality. That is why the low soil diversity seems to be crucial in the face of some contemporary problems like climate change, environment pollution and overpopulation^51,63^. If resources of semi-natural soils in the cities cannot be saved, it is necessary to designate new places for urban greenery and biomass production which would help improve microclimate, resident living conditions and urban aesthetics^65–67^. Such solutions would help improve microclimate, resident living conditions and urban aesthetics. While the exploitation of each and every free space in a city is desirable practice, the principles of sustainable urban spatial development should be upheld to protect agricultural and semi-natural soils.

## Conclusions

The presented approach allowed us to prepare both spatial and temporal analysis of the urban pedodiversity. We demonstrated that pedodiversity index (PI) values are highly spatially and temporally variable and are associated with urban development and changes in the city’s internal structure. It was revealed that land and soil cover changes significantly affected pedodiversity in the city of Inowrocław. First and foremost, between 1934 and 1978 (the period of intensive city development) the most heavily transformed areas were created and spread. This was mainly the result of the rapid development of industry and the transformation of arable land into housing. Those transformations increased the pedodiversity index within the developing areas (formation of technogenic soils). In the next period (1978–2016), restructuring of the economy and the specific development of residential areas took place which favoured a much slower increase in pedodiversity. Regardless, during both periods a different trend was observed. The discontinuous urban fabric was transforming into denser, more continuous fabric, resulting in decreasing fragmentation.

We proved that a big advantage of the presented approach is that it objectively analyses the pedodiversity of any urban area using both qualitative and quantitative methods. The method can be used in landscape monitoring and in determining ecosystem services in urban and landscape planning and environmental management. However, we realise that the method might be revised with additional landscape metrics or the application of the method in different study areas. Moreover, the presented research can be enriched with a comparison of the method using an approach aimed to calculate pedodiversity, geodiversity, and biodiversity within the same selected area.

## Supplementary Information

Supplementary Information 1.

Supplementary Information 2.

Supplementary Information 3.

## Data Availability

The data that support our research are available from the corresponding authors upon a reasonable request.
