# Supplementary material for: The influence of city development on urban pedodiversity
Source: Sci Rep. 2022 Apr 9;12:6009. doi: 10.1038/s41598-022-09903-5 (PMC8994749; doi:10.1038/s41598-022-09903-5)
Supplement: Supplementary file 1 — Supplementary Information 1. [file 41598_2022_9903_MOESM1_ESM.pdf]

Statistical analysis results used for reduction of variables

I. Kaiser–Mayer–Olkin measure of sampling adequacy and Bartlett's test of sphericity

Kaiser–Mayer–Olkin and Bartlett's tests		**2016**	**1978**	**1934**
KMO measure of sampling adequacy		0.587	0.598	0.609
Bartlett's test of sphericity	approximated Chi-squared	72,638.594	72,567.868	72,567.868
	df intrinsic accuracy	28	28	28
	significance	0	0	0

II. Spearman's rank correlation

	**2016**							
	PR	NUMP	SHDI	SIDI	SHEI	SIEI	Hmax	TE
PR	1.000	0.807	0.852	0.810	0.657	**0.708**	**1.000**	**0.746**
NUMP	0.807	1.000	0.783	0.754	0.669	0.690	0.807	0.947
SHDI	0.852	0.783	1.000	0.993	0.939	0.958	0.852	0.787
SIDI	0.810	0.754	0.993	1.000	0.960	0.981	0.810	0.762
SHEI	0.657	0.669	0.939	0.960	1.000	0.991	0.657	0.706
SIEI	**0.708**	0.690	0.958	0.981	0.991	1.000	0.708	**0.715**
Hmax	**1.000**	0.807	0.852	0.810	0.657	0.708	1.000	0.746
TE	**0.746**	0.947	0.787	0.762	0.706	**0.715**	0.746	1.000
	**1978**							
	NUMP	PR	Hmax	TE	SHDI	SIDI	SHEI	SIEI
NUMP	1.000	0.867	0.867	0.931	0.820	0.792	0.710	0.732
PR	0.867	1.000	**1.000**	**0.795**	0.867	0.831	0.706	**0.746**
Hmax	0.867	**1.000**	1.000	0.795	0.867	0.831	0.706	0.746
TE	0.931	**0.795**	0.795	1.000	0.824	0.802	0.751	**0.761**
SHDI	0.820	0.867	0.867	0.824	1.000	0.994	0.954	0.968
SIDI	0.792	0.831	0.831	0.802	0.994	1.000	0.971	0.985
SHEI	0.710	0.706	0.706	0.751	0.954	0.971	1.000	0.994
SIEI	0.732	**0.746**	0.746	**0.761**	0.968	0.985	0.994	1.000
	**1934**							
	PR	NUMP	Hmax	SHDI	SIDI	SIEI	TE	SHEI
PR	1.000	0.934	**1.000**	0.901	0.884	**0.837**	**0.889**	0.814
NUMP	0.934	1.000	0.934	0.865	0.849	0.810	0.956	0.794
Hmax	**1.000**	0.934	1.000	0.903	0.884	0.837	0.888	0.814
SHDI	0.903	0.865	0.901	1.000	0.998	0.987	0.881	0.979
SIDI	0.884	0.849	0.884	0.998	1.000	0.993	0.869	0.987
SIEI	**0.837**	0.810	0.837	0.987	0.993	1.000	**0.841**	0.998
TE	**0.889**	0.956	0.888	0.881	0.869	**0.841**	1.000	0.831
SHEI	0.814	0.794	0.814	0.979	0.987	0.998	0.831	1.000

III. Principal Component Analysis

2016				
	Axis 1	Axis 2	Axis 3	Axis 4
Eigenvalues	26.828	0.948	0.594	0.104
Percentage	93.844	3.316	2.077	0.362
Cumul. Percentage	93.844	97.160	99.240	99.600
PCA variable loadings				
	Axis 1	Axis 2	Axis 3	Axis 4
NUMP	0.336	-0.358	0.521	-0.108
PR	0.476	-0.384	-0.484	-0.136
SHDI	0.311	0.292	-0.011	-0.558
SIDI	0.302	0.382	0.005	-0.41
SHEI	0.337	0.408	0.036	0.619
Hmax	0.409	-0.283	-0.36	0.269
SIEI	0.312	0.450	0.026	0.160
TE	0.306	-0.211	0.602	0.095
**1978**				
	Axis 1	Axis 2	Axis 3	Axis 4
Eigenvalues	24.347	1.017	0.430	0.103
Percentage	93.523	3.906	1.653	0.394
Cumul. Percentage	93.523	97.429	99.082	99.476
PCA variable loadings				
	Axis 1	Axis 2	Axis 3	Axis 4
NUMP	0.370	-0.331	-0.461	-0.202
PR	0.482	-0.414	0.456	-0.147
Hmax	0.399	-0.282	0.336	0.316
TE	0.310	-0.138	-0.681	0.201
SHDI	0.303	0.301	0.012	-0.526
SIDI	0.294	0.393	0.023	-0.400
SHEI	0.326	0.409	0.027	0.583
SIEI	0.302	0.456	0.03	0.146
**1934**				
	Axis 1	Axis 2	Axis 3	Axis 4
Eigenvalues	18.341	1.178	0.340	0.105
Percentage	91.190	5.858	1.688	0.520
Cumul. Percentage	91.190	97.048	98.736	99.256
PCA variable loadings				
	Axis 1	Axis 2	Axis 3	Axis 4
PR	0.508	-0.414	-0.541	-0.252
NUMP	0.393	-0.351	0.387	-0.373
Hmax	0.374	-0.214	-0.203	0.695
SHDI	0.296	0.323	0.008	-0.287
SIDI	0.285	0.404	-0.019	-0.276
SIEI	0.288	0.458	-0.044	0.032
TE	0.316	-0.128	0.716	0.222
SHEI	0.308	0.407	-0.036	0.323
