# Supplementary material for: The influence of city development on urban pedodiversity
Source: Sci Rep. 2022 Apr 9;12:6009. doi: 10.1038/s41598-022-09903-5 (PMC8994749; doi:10.1038/s41598-022-09903-5)

I. Results for 1934

ANOVA

Test for equal means					
	Sum of squares	df	Mean square	F	p (same)
Between groups:	77,055,700	7	11,008,000	3,303	0
Within groups:	85,423,000	25,632	3,332.67	Permutation p (n=99,999)	
Total:	162,479,000	25,639	0.00		
Components of variance (only for random effects):					
Var(group): 3,433.58	Var(error): 3,332.67			ICC: 0.507457	
omega2:	0.4741				
Levene´s test for homogeneity of variance, from means	p (same):	0			
Levene´s test, from medians	p (same):	0			
Welch F test in the case of unequal variances: F=3,062, df=1.075E04, p=0					

Kruskal–Wallis test for equal medians

H (chi2):	12,250
Hc (tie corrected):	12,420
p (same):	0

There is a significant difference between sample medians.

Dunn’s *Post-Hoc*

	PR	NUMP	HMAX	SHDI	SIDI	SIEI	TE	SHEI
PR		< 0.001	0	0	0	0	< 0.001	0
NUMP	< 0.001		0	0	0	0	< 0.001	0
HMAX	0	0		< 0.001	< 0.001	< 0.001	< 0.001	< 0.001
SHDI	0	0	< 0.001		< 0.001	0.042	0	0.789
SIDI	0	0	< 0.001	< 0.001		< 0.001	0	< 0.001
SIEI	0	0	< 0.001	0.042	< 0.001		0	0.078
TE	< 0.001	< 0.001	< 0.001	0	0	0		0
SHEI	0	0	< 0.001	0,7887	< 0.001	0.078	0	

II. Results for 1978

ANOVA

Test for equal means					
	Sum of squares	Df	Mean square	F	p (same)
Between groups:	235,858,000	7	33,694,000	5,057	0
Within groups:	170,795,000	25,632	6,663.35	Permutation p (n=99,999)	
Total:	406,653,000	25,639	0.00		
Components of variance (only for random effects):					
Var(group): 10,510.90	Var(error): 6,663.35			ICC: 0.61	
omega2:	0.58				
Levene´s test for homogeneity of variance, from means	p (same):	0			
Levene´s test, from medians	p (same):	0			
Welch F test in the case of unequal variances: F=3,964, df=1.077E04, p=0					

Kruskal–Wallis test for equal medians

H (chi2):	14,640
Hc (tie corrected):	14,690
p (same):	0

There is a significant difference between sample medians.

Dunn’s *Post-Hoc*

	PR	NUMP	HMAX	SHDI	SIDI	SIEI	TE	SHEI
PR		0	0	0	0	0	0	0
NUMP	0		0	0	0	0	0.3964	0
HMAX	0	0		0	0	0	0	0
SHDI	0	0	0		0	0	0	< 0.001
SIDI	0	0	0	0		0	0	0
SIEI	0	0	0	0	0		0	0.2158
TE	0	0.3964	0	0	0	0		0
SHEI	0	0	0	< 0.001	0	0.2158	0	

III. Results for 2016

ANOVA

Test for equal means					
	Sum of squares	df	Mean square	F	p (same)
Between groups:	337,703,000	7	48,243,400	5,739	0
Within groups:	215,483,000	25,632	8,406.80	Permutation p (n=99,999)	
Total:	553,187,000	25,639	0.00		
Components of variance (only for random effects):					
Var(group): 15,049.90	Var(error): 8,406.80			ICC: 0.61	
omega2:	0.61				
Levene´s test for homogeneity of variance, from means	p (same):	0			
Levene´s test, from medians	p (same):	0			
Welch F test in the case of unequal variances: F=4,201, df=1.078E04, p=0					

Kruskal–Wallis test for equal medians

H (chi2):	15,520
Hc (tie corrected):	15,550
p (same):	0

There is a significant difference between sample medians.

Dunn’s *Post-Hoc*

	PR	NUMP	HMAX	SHDI	SIDI	SIEI	TE	SHEI
PR		0	0	0	0	0	0	0
NUMP	0		0	0	0	0	< 0.001	0
HMAX	0	0		0	0	0	0	0
SHDI	0	0	0		0	0	0	0
SIDI	0	0	0	0		0	0	0
SIEI	0	0	0	0	0		0.5259	0
TE	0	< 0.001	0	0	0	0.5259		0
SHEI	0	0	0	0	0	0	0	

IV. Classification tree (Ward method) for all analysed years

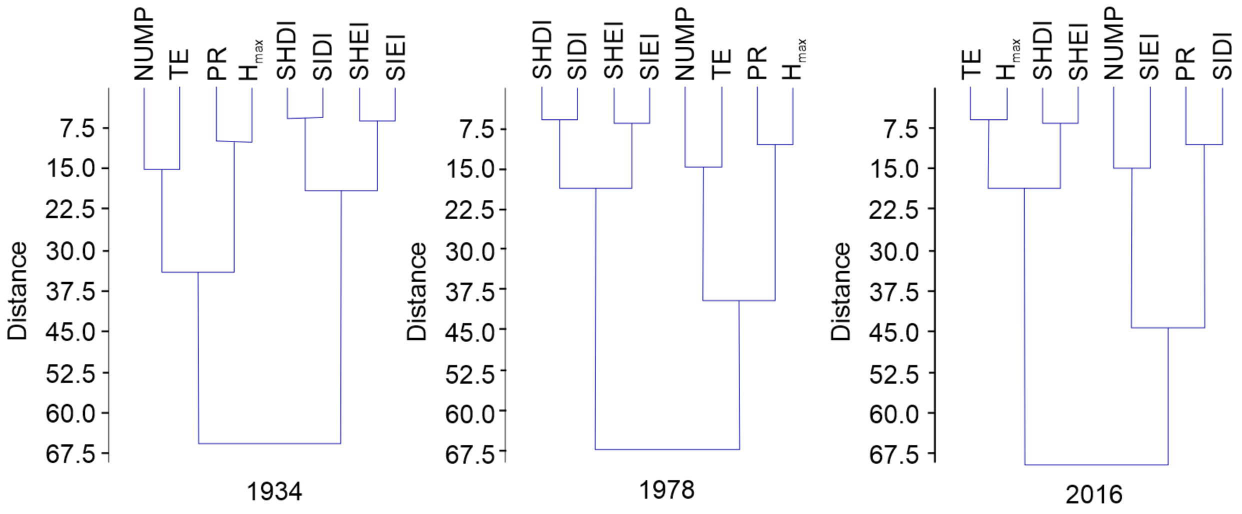

Supplement: Supplementary file 2 — Supplementary Information 2. [file 41598_2022_9903_MOESM2_ESM.pdf]
