# Supplementary material for: The influence of city development on urban pedodiversity
Source: Sci Rep. 2022 Apr 9;12:6009. doi: 10.1038/s41598-022-09903-5 (PMC8994749; doi:10.1038/s41598-022-09903-5)

I. Descriptive statistics for land cover patches and USCs patches [m^2]

	Descriptive statistics for land cover patches								
	mean	median	minimum	maximum	first quartile	third quartile	range	variance	standard deviation
2016	5088.917	880.082	0.0000268	843577.325	216.254	3458.917	843577.325	569729541.141	23869.008
1978	7564.314	1289.387	0.0000186	779971.137	315.407	4700.311	779971.137	999561143.112	31615.837
1934	20330.577	2882.097	3.796	863137.270	1030.704	10585.310	863133.474	4378666008.198	66171.489
	Descriptive statistics for urban soil complexes								
	mean	median	minimum	maximum	first quartile	third quartile	range	variance	standard deviation
2016	8678.581	2122.746	0.0000784	1575285.456	568.725	5901.810	1575285.456	1818646628.237	42645.593
1978	13162.218	2828.394	0.00108	1069857.941	936.525	8464.848	1069857.940	2547191600.239	50469.710
1934	28163.445	4151.080	0.0192	953059.168	1159.448	15854.142	953059.148	7599452554.995	87174.839

II. Descriptive statistics for landscape metric results and pedodiversity index values

	2016									
	mean	median	mode	minimum	maximum	first quartile	third quartile	range	variance	standard deviation
NUMP	5.851	5	1	1	32	3	8	31	14.570	3.817
PR	2.980	3	3	1	7	2	4	6	1.653	1.286
Hmax	0.978	1.099	1.099	0	1.946	0.693	1.386	1.946	0.260	0.510
TE	348.618	339.311	0	0	1428.90	145.72	523.13	1428.90	67237.5	259.302
SHDI	0.538	0.525	0	0	1.616	0.161	0.876	1.616	0.169	0.412
SIDI	0.298	0.276	0	0	0.791	0.070	0.514	0.791	0.055	0.235
SHEI	0.440	0.472	0	0	0.999	0.194	0.687	0.999	0.086	0.293
SIEI	0.426	0.421	0	0	0.999	0.126	0.724	0.999	0.100	0.317
PI	6.807	7	3	3	12	4	9	9	7.708	2.776
	1978									
	mean	median	mode	minimum	maximum	first quartile	third quartile	range	variance	standard deviation
NUMP	4.384	4	1	1	18	2	6	17	7.454	2.730
PR	2.760	3	3	1	7	2	4	6	1.546	1.243
Hmax	0.897	1.099	1.099	0	1.946	0.693	1.386	1.946	0.264	0.514
TE	291.337	273.577	0	0	1232.189	91.288	444.623	1232.189	53297.185	230.862
SHDI	0.462	0.382	0	0	1.687	0.095	0.787	1.687	0.156	0.394
SIDI	0.256	0.197	0	0	0.796	0.037	0.480	0.796	0.051	0.227
SHEI	0.394	0.388	0	0	0.999	0.122	0.648	0.999	0.086	0.294
SIEI	0.374	0.315	0	0	0.999	0.071	0.675	0.999	0.098	0.312
PI	6.468	6	3	3	12	4	9	9	7.683	2.772
	1934									
	mean	median	mode	minimum	maximum	first quartile	third quartile	range	variance	standard deviation
PR	2.199	2	2	1	6	1	3	5	1.192	1.092
NUMP	3.238	3	1	1	14	1	4	13	5.073	2.252
Hmax	0.663	0.693	0.693	0	1.792	0	1.099	1.792	0.258	0.508
SHDI	0.306	0.177	0	0	1.622	0	0.538	1.622	0.121	0.348
SIDI	0.171	0.080	0	0	0.786	0	0.301	0.786	0.041	0.204
SIEI	0.267	0.148	0	0	0.999	0	0.484	0.999	0.091	0.302
TE	166.751	154.212	0	0	818.677	0	269.286	818.677	26654.490	163.262
SHEI	0.296	0.224	0	0	0.999	0	0.536	0.999	0.089	0.298
PI	6.124	6	3	3	12	3	8	9	7.924	2.815

III. Pedodiversity index changes: balance between analysed years

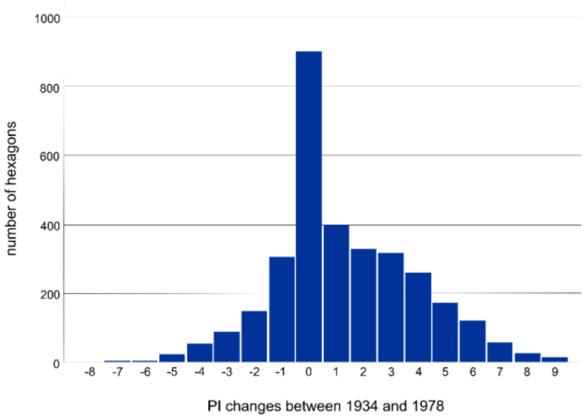

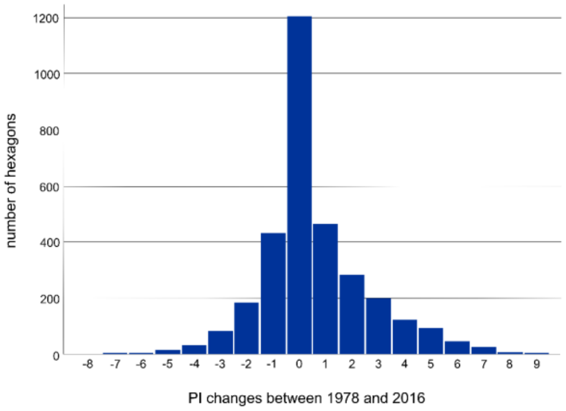

Supplement: Supplementary file 3 — Supplementary Information 3. [file 41598_2022_9903_MOESM3_ESM.pdf]
